# Fever of unknown origin (FUO): a 7-year clinical experience, etiological distribution, and diagnostic approaches

**DOI:** 10.1186/s12879-025-11979-z

**Published:** 2025-11-20

**Authors:** Meryem Sena Kaya, Sibel Yıldız Kaya, Rıdvan Karaali, Günay Can, İlker İnanç Balkan, Bilgül Mete, Fehmi Tabak, Neşe Saltoğlu

**Affiliations:** 1 Department of Infectious Diseases and Clinical Microbiology, Istanbul University-Cerrahpasa, Cerrahpasa Medical Faculty, Istanbul, TR-34300 Türkiye; 2 Department of Public Health, Istanbul University-Cerrahpasa, Cerrahpasa Medical Faculty, Istanbul, TR-34300 Türkiye

**Keywords:** FUO, Tuberculosis, Vasculitis, Malignancy, COVID-19, Immunosuppression, HIV, Recurrent fever, PET-CT

## Abstract

**Background:**

Fever of unknown origin (FUO) remains a significant diagnostic challenge. Changes in patient populations and diagnostic technologies may shift the spectrum of underlying etiologies. This study aimed to examine recent FUO cases at a single tertiary center to identify changes in etiology compared with previous reports and between the pre- and post-COVID-19 pandemic periods, assess the diagnostic value of laboratory and imaging findings, and examine FUO characteristics in underrepresented subpopulations, including people living with HIV (human immunodefficiency virus), those on immunosuppressive therapy, and individuals with recurrent fever, with the goal of informing a center-specific diagnostic approach.

**Methods:**

A retrospective analysis was performed on 100 patients hospitalized with FUO between 2017 and 2024, classified according to Durack and Street’s criteria. Subgroups included classical (*n* = 85), HIV-associated (*n* = 13), neutropenic (*n* = 1), and nosocomial FUO (*n* = 1). Clinical, laboratory, imaging, and biopsy findings were reviewed, and subgroup-specific analyses were conducted.

**Results:**

Among 100 FUO patients (mean age 45.1 years; 52 males), recurrent fever was observed in 14 patients within the classical FUO group. The median fever duration at admission was 6 weeks. The average time to diagnosis was 14 days, and the mean hospital stay was 21 days. Final diagnoses were: collagen vascular diseases (CVD, 39%), infections (19%), malignancies (17%), miscellaneous (12%), and undiagnosed (13%). Vasculitis predominated among CVDs (25.6%), extrapulmonary tuberculosis (TB) among infections (36.8%), lymphomas among malignancies (52.9%) and, subacute thyroiditis among “miscellaneous” category (25%). In subgroup analysis, people living with HIV had no CVD, while no infections were found in recurrent fever cases. Among people living with HIV, infections (46%) and malignancies (38%) were most common. Immunosuppressed patients had diverse etiologies, including drug-induced pneumonitis and disease flares. CRP ≥140 mg/L, low hemoglobin, and anti-HIV positivity were independent predictors of malignancy; ferritin ≥875 µg/L was significantly associated (*p* = 0.01). PET-CT (Positron Emission Tomography – Computed Tomography) had the highest diagnostic yield, especially in infections and malignancies (*p* = 0.004). Microbiological studies were diagnostic in > 50% of infectious cases; autoantibodies aided diagnosis in ~50% of CVDs. During follow-up, 11 patients died, including 7 from the malignancy group.

**Conclusion:**

Our findings indicate a shift toward non-infectious inflammatory causes of FUO, with CVD surpassing infections. PET-CT and targeted biopsies are valuable diagnostic tools, particularly in suspected malignancies or infections. Subgroup-specific approaches are essential, especially in people living with HIV and immunosuppressed patients. Despite advances, a proportion of FUO cases remain undiagnosed, underscoring the need for stepwise, individualized diagnostic strategies.

**Clinical trial number:**

Not applicable.

## Introduction

Fever of unknown origin (FUO) was initially conceptualized by Petersdorf and Beeson in 1961 as a fever of ≥38,3 °C persisting for at least three weeks, with no established diagnosis despite a minimum of one week of inpatient evaluation [1]. In 1991, Durack and Street refined this definition by classifying FUO into four major subtypes—classical, nosocomial, neutropenic, and HIV-related—and by modifying the diagnostic threshold to include either three outpatient visits or three days of hospitalization with inconclusive microbiological work-up, including at least 48 hours of culture-based studies [2]. Subsequently, de Kleijn and colleagues proposed a more structured minimum diagnostic protocol that combined both quantitative and qualitative parameters [3]. In 2003, Knockaert et al. further advanced FUO categorization by emphasizing five overarching etiological groups [4]. The most recent Delphi Consensus Report, published in 2024, reaffirmed the relevance and applicability of this five-group classification framework [5].

Despite advances in microbiological and imaging techniques, FUO remains diagnostically challenging, with 7–30% of cases remaining unexplained [3, 6]. The inability to apply a universal algorithm for FUO diagnosis is primarily due to the wide range of potential etiologies and the influence of demographic factors such as age, geographic location, and immune status, all of which impact the diagnostic approach. The etiological spectrum of classical FUO is generally categorized into five major groups: infectious diseases, malignancies, collagen vascular diseases, miscellaneous conditions, and cases that remain undiagnosed [4, 5].

Infectious causes—especially tuberculosis—remain prevalent in low- and middle-income countries, while non-infectious inflammatory diseases and malignancies are more common in high-income settings [7–9]. Despite an overall increase in infectious diseases among causes of FUO in China, the rates attributed to tuberculosis have decreased. Rates attributed to both autoimmune and neoplastic diseases have also decreased [10].

Age is also a determinant, with autoinflammatory conditions seen in younger adults and vasculitides like giant cell arteritis in older populations [4, 11].

In recent years, the widespread use of PET-CT, immunosuppressive therapies, and molecular diagnostics has reshaped the diagnostic landscape. Consideration of individual and regional characteristics, based on the standard diagnostic approach, may be helpful in determining the cause of FUO [8, 12–15].

This study aims to retrospectively analyze a cohort of hospitalized patients diagnosed with FUO. The primary objectives are to characterize the etiological profile of FUO in recent years and to assess changes over time, including those after the COVID-19 pandemic, evaluate the diagnostic contribution of laboratory analyses, imaging modalities and invasive procedures. Additionally, this study explores FUO characteristics in underrepresented subpopulations in FUO studies, such as people living HIV, those receiving immunosuppressive therapy, and individuals presenting with recurrent fever episodes. The findings are expected to support the development of a center-specific, evidence-based diagnostic algorithm aimed at reducing diagnostic delays and unnecessary testing in clinical practice.

## Materials and methods

**Fig. 1:**
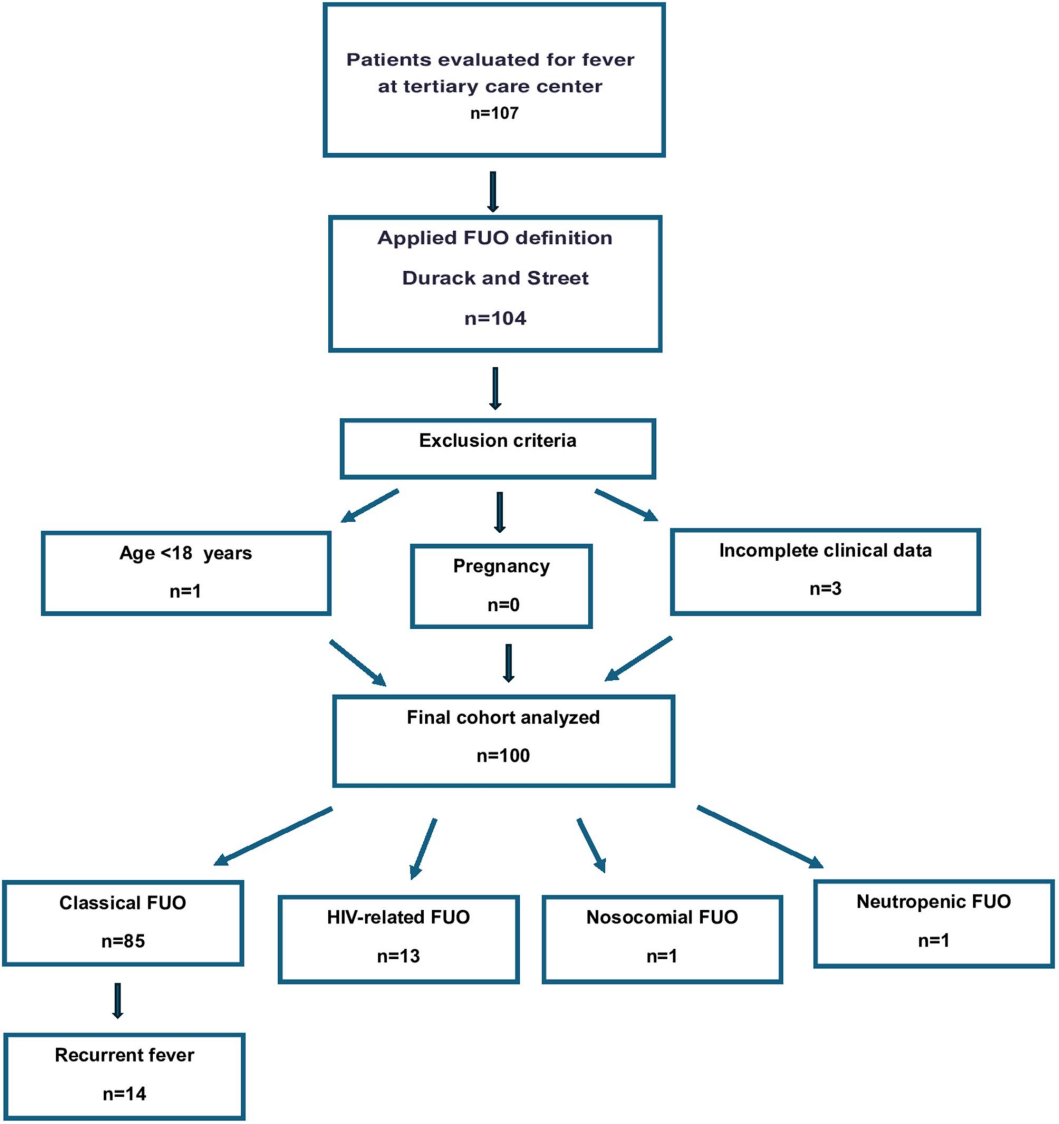
Flow diagram of patient selection

We retrospectively analyzed a seven-year cohort of patients managed for FUO at our tertiary care center between May 2017 and June 2024. This study was conducted at Istanbul University-Cerrahpasa, Cerrahpasa Medical Faculty Hospital, a tertiary care hospital located in İstanbul/Türkiye. The definition of FUO used in this study was based on the criteria proposed by Durack and Street, which include a body temperature of ≥38,3 °C persisting for at least three weeks, and failure to reach a diagnosis despite three outpatient visits or three days of inpatient evaluation, including at least 48 hours of microbiological investigations [2]. In addition to classical FUO, patients classified as HIV-related, neutropenic, or nosocomial FUO were also included. Recurrent FUO, a subgroup of classical FUO, defined by at least two episodes of fever of limited duration, with minimal fever-free periods of at least 2 days to 2 weeks depending on definitions, and apparent remission of the symptoms [16]. A total of 100 cases meeting these criteria were enrolled in the study. Patients under 18 years of age, pregnant women, and patients with incomplete clinical data were excluded (see Fig. 1).

For each patient, demographic and clinical data were recorded, including age, sex, presenting symptoms, duration of hospitalization, comorbid conditions, and the time interval between fever onset and hospital admission. Physical examination findings at admission—such as lymphadenopathy, hepatosplenomegaly, skin lesions, and joint involvement—were also documented. Laboratory investigations encompassed complete blood count, peripheral blood smear, erythrocyte sedimentation rate (ESR), renal function tests, liver enzymes such as aspartate aminotransferase (AST), alanine aminotransferase (ALT), alkaline phosphotase (ALP), Gamma-glutamyl transferase (GGT), lactate dehydrogenase (LDH), ferritin, C-reactive protein (CRP), and procalcitonin. Results from microbiological cultures, viral serologies, and imaging modalities—including chest X-ray, computed tomography (CT), magnetic resonance imaging (MRI), PET-CT, abdominal ultrasonography, transthoracic and transesophageal echocardiography—were collected. Data from invasive diagnostic procedures, such as tissue biopsies, colonoscopy, gastroscopy, lumbar puncture, thoracentesis, paracentesis, and bronchoscopy, were also included.

Patients were categorized into five diagnostic groups based on their final diagnosis: infectious diseases, malignancies, collagen vascular diseases, miscellaneous conditions, and undiagnosed cases. Imaging findings were reviewed in the context of the final diagnosis. If the imaging modality contributed directly to establishing the diagnosis, it was labeled “diagnostically useful.” Findings that were incidental and not related to the final diagnosis were classified as “not diagnostic.”

Prognostic assessment was conducted using outpatient follow-up records and e-Nabız Personal Health Record System, a national electronic medical registry provided by the Ministry of Health. Outcomes were categorized as survival or death, with a minimum follow-up period of six months after hospital discharge. For patients with no available follow-up data, outcomes were recorded as “data unavailable.”

Statistical analyses were performed using IBM SPSS Statistics for Windows, version 26.0. Categorical variables were compared using the chi-square test, while continuous variables were analyzed with one-way ANOVA and Tukey HSD or the Kruskal–Wallis test, depending on distribution. Receiver operating characteristic (ROC) analysis was used to assess the predictive value of CRP and ferritin levels for malignancy. Variables found to be significant in univariate analysis were included in a binary logistic regression model to identify independent predictors of malignancy. A two-tailed p-value of < 0.05 was considered statistically significant.

This study was approved by the Ethics Committee of Istanbul University-Cerrahpaşa on February 12, 2025 (Approval No: 1,228,704). All procedures were performed in accordance with the ethical standards of the institutional and/or national research committee, as well as the principles outlined in the Declaration of Helsinki (1964) and its subsequent revisions or equivalent ethical guidelines.

## Results

**Fig. 2:**
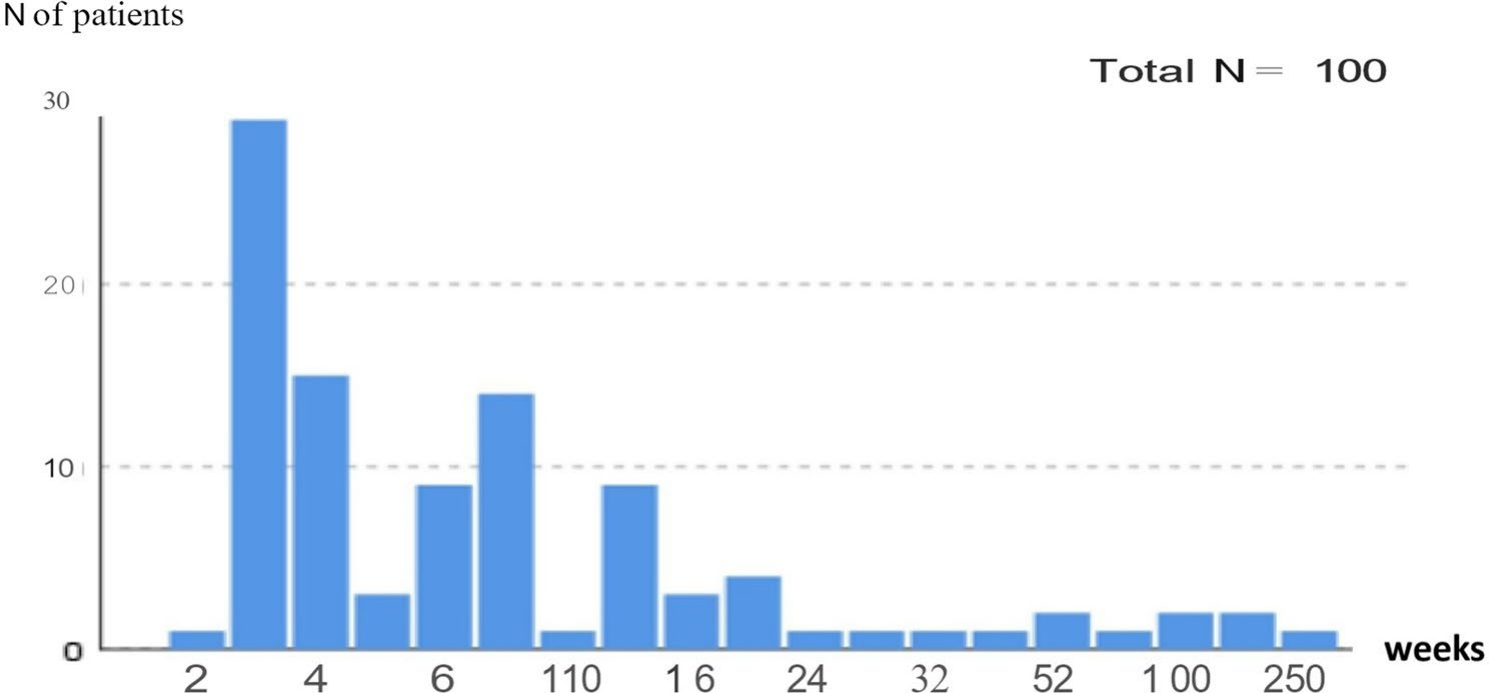
Distribution of fever duration of cases (1 case had a fever duration of 2 weeks due to nosocomial FUO)

A total of 100 patients diagnosed with FUO and hospitalized in our department, comprising 52 males and 48 females. The mean age was 45.07 ± 17.52 years (range: 20–88), with no statistically significant difference between genders (43.94 ± 16.50 for males vs. 46.29 ± 18.61 for females; *p* = 0.50) (Gender and age distribution of patients among diagnostic groups are presented in the Table 1). The median time from fever onset to hospital admission was six weeks (Fig. 2 shows the distribution of fever duration at the time of admission among the cases). While time to diagnosis did not differ significantly between groups, the mean hospital stay was significantly longer in the malignancy group (*p* = 0.003).

**Table 1:**
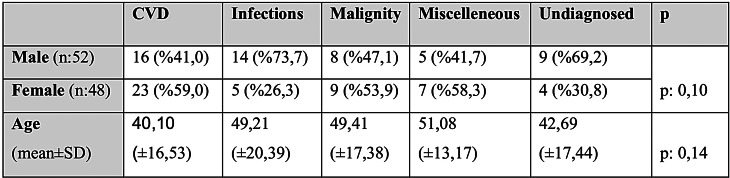
Age and gender distribution according to diagnosis groups

It was observed that 96% of cases used antibiotics before admission to our hospital, and 29% of cases were examined in an external center before our clinic.

**Fig. 3:**
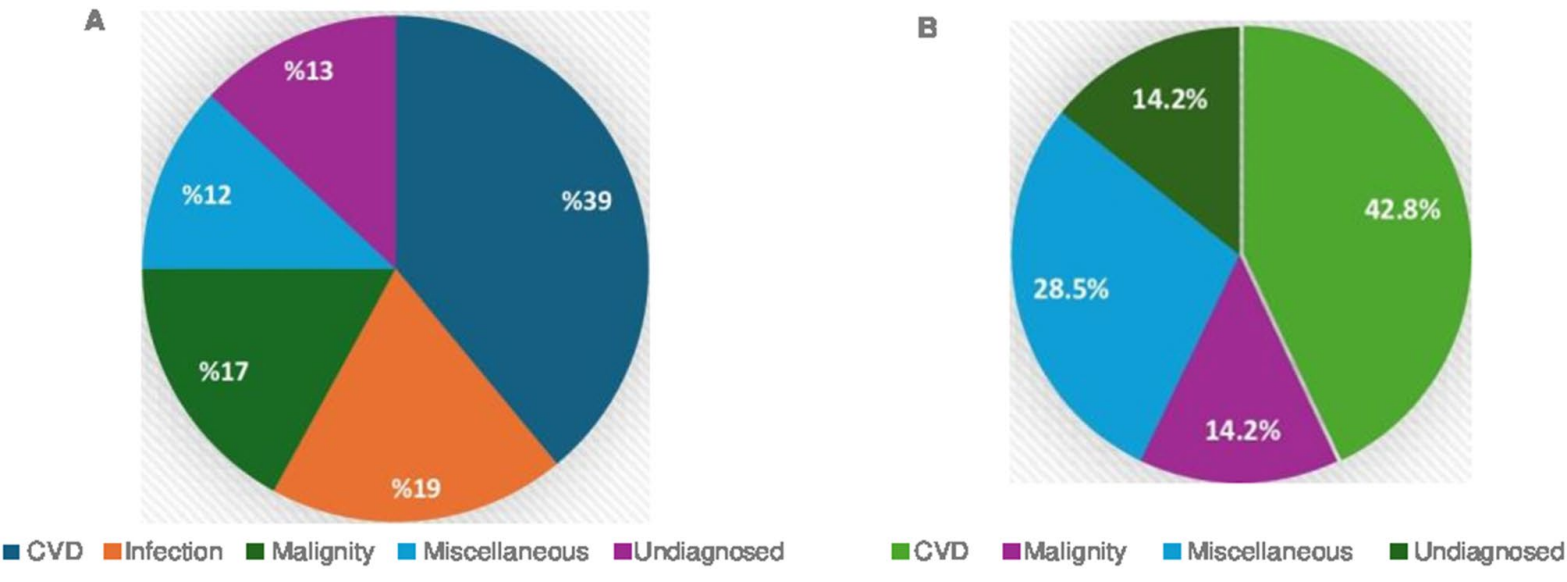
Diagnostic distribution of all FUO cases (**A**) and recurrent fever cases (**B**). (CVD: Collagen Vascular Disease)

According to the Durack and Street classification, 85 cases were identified as classical FUO, 13 as HIV-associated FUO, 1 as neutropenic FUO, and 1 as nosocomial FUO. Fourteen patients in the classical FUO group presented with a recurrent fever pattern. Final diagnoses were established as follows: CVD in 39 cases, infectious diseases in 19, malignancies in 17, other conditions in 12, and undiagnosed FUO in 13 cases (Fig. 3). There were no significant differences among these groups in terms of age or sex distribution.

In the CVD group, the most common conditions were vasculitis (25.6%, *n* = 10) and adult-onset Still’s disease (23.0%, *n* = 9). TB (36.8%) predominated in the infectious disease group, while lymphomas accounted for 52.9% of the malignancies (29.4% non-Hodgkin, 23.5% Hodgkin lymphoma). Subacute thyroiditis was the most frequent diagnosis in the “other” category (25%). The diagnostic distribution is presented in Table 2.

**Table 2:**
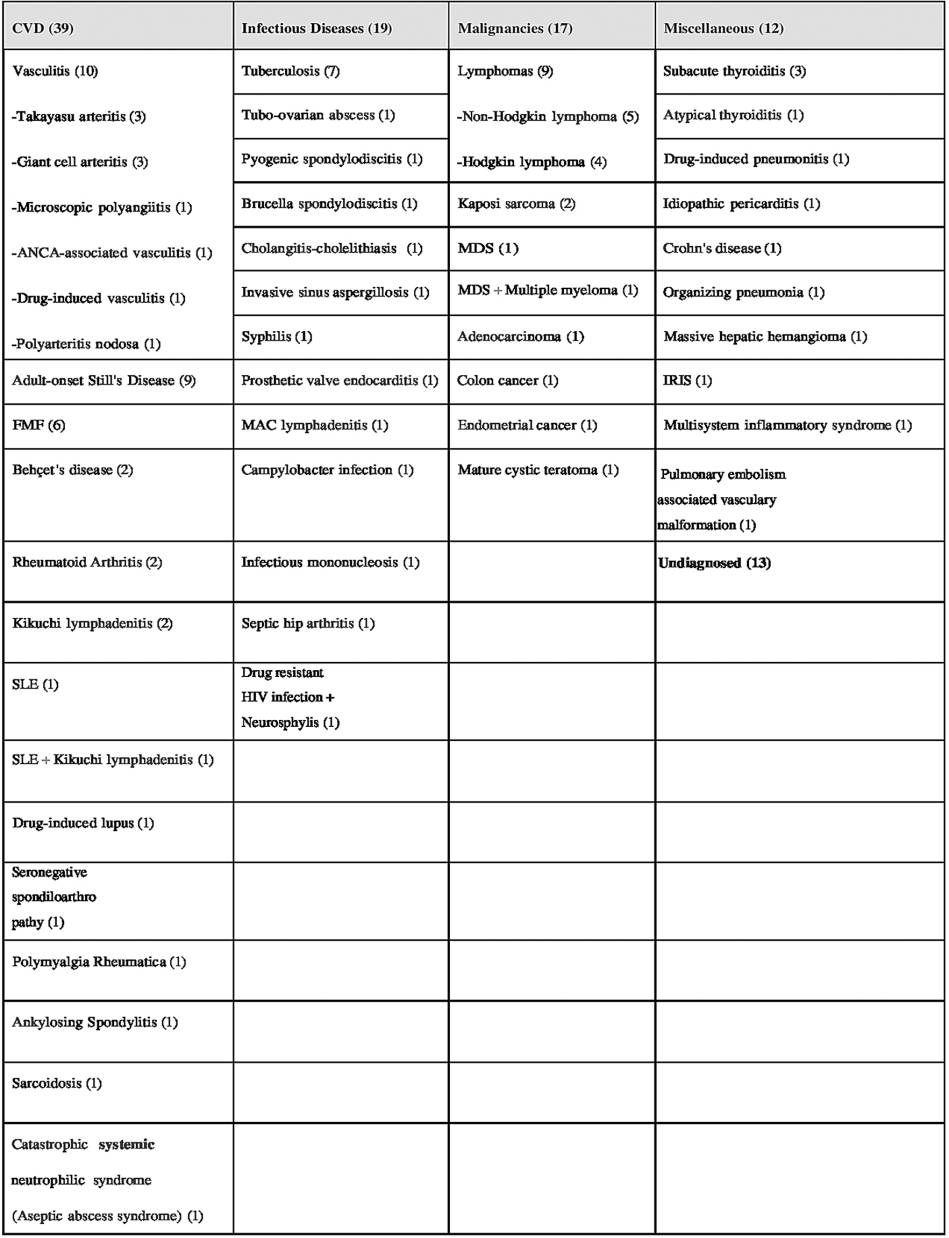
Etiology of FUO cases according to diagnostic groups

A total of 23 patients had immunosuppressive conditions, including 13 people living with HIV and 10 receiving immunosuppressive therapy. Among people living with HIV, 76.9% (*n* = 10) had acquired immunodeficiency syndrome (AIDS, CD4 < 200/mm^3^). Of these, 6 (46.1%) were diagnosed with infections (e.g. disseminated TB, syphilis, mycobacterium avium complex (MAC)), 5 (38.4%) with malignancy (Kaposi’s sarcoma or lymphoma), 1 (7.6%) with miscellaneous diagnosis group, and 1 remained undiagnosed. In one patient in the miscellaneous group the cause of fever was thought to be immune reconstitution inflammatory syndrome (IRIS) and he was discharged with healing after following up with systemic steroid treatment. None had CVD. The mortality rate in the HIV group was 23% (3/13; 1 TB, 1 lymphoma, 1 undiagnosed).

Among patients on immunosuppressive therapy (*n* = 10), diagnoses included CVD (*n* = 4), malignancy (*n* = 1), infection (*n* = 1), other causes (*n* = 3), and undiagnosed (*n* = 1). Two CVD cases were attributed to underlying disease exacerbation. Two patients were diagnosed with drug-induced pneumonitis (ocrelizumab, methotrexate). No deaths occurred in this subgroup.

Fourteen patients were admitted with recurrent fever; 6 (42.8%) were diagnosed with CVD, 2 (14.2%) with malignancy, 4 (28.5%) with other causes, and 2 (14.2%) remained undiagnosed. No infectious etiology was identified in this subgroup. Two patients in this group (both lymphoma) died during follow-up (14.2%) (Fig. 3).

Arthralgia at presentation was significantly more common in CVD patients (46.1%) than in other diagnostic groups (*p* = 0.001), while weight loss was less frequent in CVD cases (*p* = 0.042). Fatigue was significantly more common among patients with malignancy or undiagnosed FUO (*p* = 0.044) (Table 3). No significant intergroup differences were observed in the rates of lymphadenopathy, hepatosplenomegaly, arthritis, or rash.

**Table 3:**
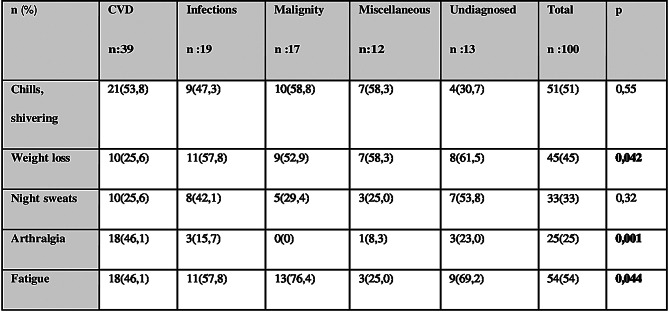
Evaluation of symptoms at admission of FUO cases according to diagnostic groups

**Table 4:**
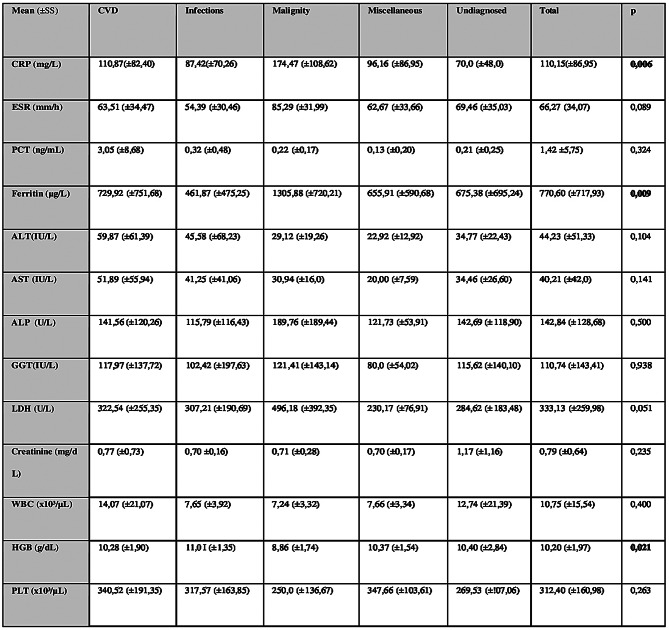
Laboratory findings of patients at admission according to FUO diagnostic groups

Laboratory analysis revealed significantly elevated CRP and ferritin levels, and lower hemoglobin concentrations in the malignancy group (see Table 4). ROC analysis showed that a CRP level ≥140 mg/L (AUC = 0,712, CI = 0,55–0,87) was predictive of malignancy (*p* = 0.006), and a ferritin level ≥875 μg/L (AUC = 0.753, CI = 0.63–0.88) was significantly associated with malignancy (*p* = 0.01) (see Table 5). In multivariate logistic regression, low hemoglobin, CRP ≥140 mg/L, and anti-HIV positivity were identified as independent predictors of malignancy (see Table 6).

**Table 5:**
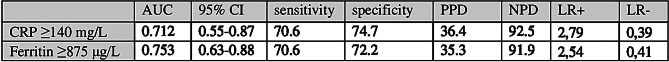
Diagnostic values of CRP and Ferritin according to the cut-off points of the ROC curve

**Table 6:**
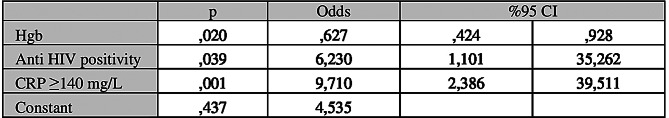
Multivariate logistic regression analysis

The overall diagnostic yield of microbiological tests (culture positivity (31.5%), serology, polymerase chain reaction (PCR)) was 57.8% among infectious cases. Mycobacteria were isolated in culture in 2 cases (1 MAC, 1 TB). In one TB case, the diagnosis was made by PCR positivity from the tissue. CMV Ig-M and Ig-G positivity in one case, VDRL positivity in 2 cases, and Rose-Bengal and Wright agglutination tests in 1 case contributed to the diagnosis.

Autoantibody positivity was identified in 16 CVD cases and contributed to diagnosis in 7 patients. Ophthalmologic examination aided diagnosis in 6 of 78 patients (7.6%), including cases of spondyloarthritis, temporal arteritis, and Takayasu’s arteritis. In addition to the CVD cases, ophthalmologic findings contributed to the diagnosis in three other patients: two with ocular involvement of syphilis and one with a choroidal tubercle.

Echocardiography contributed to diagnosis in 5 patients: pericardial involvement (*n* = 3; CVD), endocarditis (*n* = 1; infections), and pericarditis (*n* = 1; miscellaneous group diseases).

Among imaging modalities, abdominal ultrasonography, abdominal CT, and thoracic CT contributed to diagnosis in 29.3%, 26.3%, and 30.8% of cases, respectively, without significant differences between diagnostic groups. PET-CT, performed in 82 patients, was diagnostically helpful in 59.7% of cases, with a statistically significant contribution in the infection and malignancy groups (*p* = 0.004; Table 7).

**Table 7:**
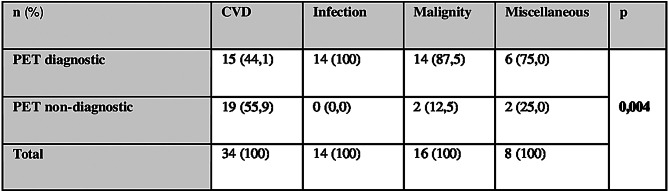
Contribution of PET-CT results to diagnosis according to FUO etiologies

**Fig. 4:**
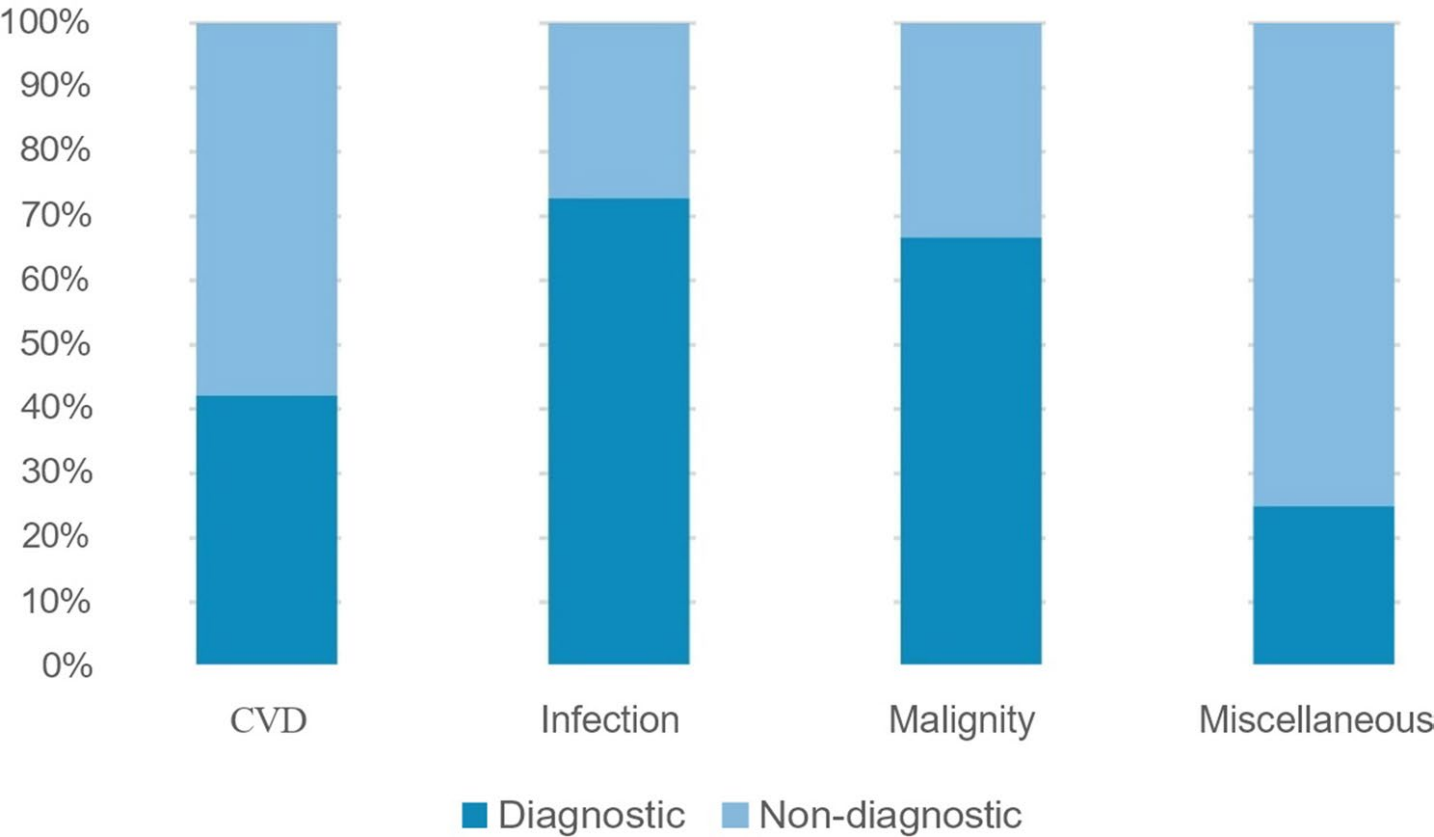
Comparison of the contribution of biopsy to diagnosis between etiologies

Biopsies contributed to diagnosis in 48.2% (28/58) of all procedures, including 48% (12/25) of lymph node biopsies and 20% (4/20) of bone marrow biopsies. While diagnostic yield was higher in the infection and malignancy groups, this difference did not reach statistical significance (*p* = 0.267) (Fig. 4 presents the intergroup differences in the diagnostic contribution of biopsy.).

Of 98 patients with available post-discharge data, 11 died during follow-up. These included 7 patients with malignancies (two-thirds of deaths), 2 with disseminated TB, and 2 undiagnosed cases. The mortality rate among undiagnosed patients was 15.3%.

Among 100 FUO cases, one was classified as neutropenic fever and another as nosocomial fever. In the nosocomial case, further evaluation revealed the fever was related to the underlying colorectal malignancy. The patient died during follow-up. The neutropenic fever case also resulted in death in the second week of hospitalization, before a definitive diagnosis could be established.

No significant differences were observed in etiological distribution between the pre-COVID-19 pandemic (*n* = 47) and post-pandemic (*n* = 53) periods (*p* = 0.128).

## Discussion

This study presents a comprehensive analysis of FUO cases over a seven-year period in a tertiary care center, highlighting both classical and underrepresented subgroups such as HIV-associated, immunosuppressed, and relapsing fever cases. Our findings demonstrate a dynamic shift in the etiological spectrum of FUO, with CVD emerging as the leading cause, surpassing infections, which were traditionally dominant [14, 17–19].

This trend appears consistent with data from high-income settings, where widespread immunization, timely access to healthcare, and advanced microbiological diagnostics have contributed to a relative decline in infectious etiologies. In contrast, infections remain the primary cause of FUO in low-resource regions, where diagnostic limitations persist [6, 14, 20, 21]. Interestingly, while national studies from our country previously reported infections as the most frequent cause of FUO (ranging from 34% to 68%) [18, 19, 22], our data showed a predominance of collagen vascular diseases (39%) and a relatively lower infection rate (19%). This may reflect referral bias toward more diagnostically complex patients, as well as improved access to advanced imaging and serologic testing.

In our cohort, vasculitides (27.7%) and adult-onset Still’s disease (25%) were most prevalent within the CVD group, consistent with earlier findings [20, 23]. In the diagnosis of giant cell arteritis, Doppler ultrasonography is considered nearly equivalent to temporal artery biopsy, and in the presence of characteristic findings, treatment can be initiated without histopathological confirmation. Recent updates from the American College of Rheumatology/European League Against Rheumatism (ACR/EULAR) classification criteria for giant cell arteritis emphasize the diagnostic value of temporal artery Doppler ultrasonography. This imaging modality is now considered one of the five core criteria and holds diagnostic weight comparable to temporal artery biopsy when interpreted within the appropriate clinical context [24]. Consequently, treatment initiation can be justified based on characteristic Doppler findings even in the absence of histopathological confirmation, underscoring the utility of non-invasive diagnostic approaches in GCA management.

However, due to its limited ability to visualize deep vessels such as the aorta, given its high sensitivity for detecting large vessel inflammation, PET-CT should be considered early in cases with clinical or epidemiological suspicion of large-vessel vasculitis [24].

Extrapulmonary TB (36.8%) emerged as the leading infectious etiology in this study, consistent with previous reports from both our country and international literature (%30–70) [13, 16, 19, 20]. This is largely due to its often subtle or atypical presentation and its continued endemicity in Türkiye, with an incidence of 13 per 100,000 population in 2023 [25]. Extrapulmonary forms can mimic various conditions and are frequently difficult to diagnose without invasive testing. Limited access to advanced diagnostics in many parts of the world and delayed culture results contribute to its frequent inclusion in FUO cases. PET-CT is a valuable tool in the diagnosis of disseminated or extrapulmonary TB, as it can detect metabolically active lesions not visible on conventional anatomical imaging. It aids in assessing systemic involvement and guiding biopsy from the most appropriate site. Our findings also demonstrated that PET imaging made a substantial contribution to the diagnosis of infectious diseases (Table 4).

Malignancies were the third most common cause (17%), with lymphomas—particularly non-Hodgkin lymphoma—being the predominant subtype. PET-CT was particularly useful in identifying malignant causes, with a diagnostic yield approaching 88% in this subgroup. The prognostic implications of malignancy were notable, as two-thirds of all deaths in the cohort occurred in this group.

Thyroiditis was a notable diagnosis within the miscellaneous group [22, 26]. In one case, despite normal thyroid function tests, PET-CT revealed thyroid uptake, and ultrasonography supported the diagnosis of thyroiditis. Following endocrinology consultation, the patient was diagnosed with atypical thyroiditis. This case underscores the importance of considering atypical presentations and employing a multidisciplinary approach in FUO evaluation.

One noteworthy observation was the absence of autoimmune diseases among HIV -FUO subgroup. The reduced prevalence of autoimmune diseases in people living with HIV has been attributed to several immunopathogenic mechanisms. Chronic depletion of CD4+ T cells leads to impaired adaptive immune responses, thereby limiting the autoreactive lymphocyte activity that drives autoimmunity. In addition, the shift toward a T helper-2 polarized cytokine milieu and the expansion of regulatory T cells contribute to suppression of autoreactive immune pathways. Furthermore, the persistent immune exhaustion observed in chronic HIV infection create an environment that is unfavorable for the initiation and propagation of classical autoimmune processes. Collectively, these mechanisms may explain the relatively low incidence of autoimmune diseases among people living with HIV [27, 28].

In HIV subgroup, mycobacterial infections and AIDS-related malignancies (lymphoma and Kaposi sarcoma) were the primary etiologies. This aligns with previous literature and suggests distinct pathogenetic mechanisms in immunocompromised hosts [29–31]. Similarly, among immunosuppressed patients (due to therapy), drug-induced pneumonitis and disease reactivation emerged as key etiologies. To the best of our knowledge, there are only some case reports in the literature [32, 33], specifically addressing FUO presented during immunosuppressive therapy, emphasizing the need for individualized diagnostic strategies in this population.

The intent is to highlight that people living with HIV, those on immunosuppressive therapy, and individuals with recurrent fevers are generally underrepresented in FUO studies, rather than suggesting that they are uncommon among FUO patients in clinical practice. Most large FUO cohorts explicitly exclude these populations, which limits how well study findings can be generalized to them.

Recurrent FUO, present in 14% of classical FUO patients, remains an underexplored clinical entity. Our results revealed a predominance of non-infectious causes and a diagnostic challenge, with 14% of these cases remaining unexplained despite extensive workup [16, 34].

From a clinical perspective, arthralgia was significantly associated with collagen vascular diseases, while fatigue and anemia were more prominent in malignancies. Although symptoms such as arthralgia, fatigue, and anemia were observed with higher frequency in specific FUO subgroups in our study and some other studies [22, 35], these clinical features should be interpreted with caution. Their diagnostic value may be influenced by the local epidemiology and etiological spectrum of FUO, and symptom patterns can vary significantly across different geographic regions and healthcare settings. Therefore, these findings may have limited external validity and should not be considered definitive diagnostic indicators without further validation.

Laboratory markers such as elevated CRP (≥140 mg/L) and ferritin (≥875 μg/L) were strongly associated with malignancy and may be incorporated into initial risk stratification. Notably, ferritin showed a robust predictive value in ROC analysis, supporting its use as a potential diagnostic adjunct.

Microbiological tests contributed to diagnosis in 60–80% of infection cases, yet accounted for only 11% of total diagnoses in the literature [23, 36]. Autoantibodies supported diagnosis in around 50% of collagen vascular disease cases. Among endoscopic methods, bronchoscopy had the highest diagnostic yield (30%), mirroring results from our prior institutional data [23].

Imaging played a pivotal role, with abdominal ultrasound and CT contributing diagnostically in 30% of cases. The incidence has been previously reported as between 10 and 20% [17, 23]. Due to its non-invasive nature and availability, USG may serve as the first-line modality. Fundoscopic examination provided key diagnostic clues in 7% of patients, particularly in cases of collagen vascular disease, syphilis, and TB. Fundoscopic examination has been recommended in previous studies as a valuable component of FUO evaluation, particularly due to its potential to reveal signs suggestive of infective endocarditis and systemic inflammatory diseases. It is advised that fundoscopy be performed whenever possible during the diagnostic workup [37, 38].

PET-CT demonstrates a high overall diagnostic contribution (30–70%), particularly in infection and malignancy groups [20, 23, 39, 40]. Although its utility was relatively limited in autoimmune disorders, early use of PET-CT has been associated with shorter hospital stays and reduced diagnostic delays, findings echoed in our cohort [40]. Nevertheless, while our findings also suggest potential benefits of FDG-PET, further multicenter prospective studies including control groups are warranted to definitively establish its diagnostic value.

In our cohort biopsies yielded diagnostic information in nearly 50% of the cases who underwent the procedure. Despite technological advances, invasive procedures remain indispensable when non-invasive diagnostics fail, and should not be delayed.

Despite a comprehensive diagnostic approach, 13% of cases remained undiagnosed. This figure is within the range reported in the literature (7–51%) and likely reflects the evolving nature of FUO populations, as common infectious causes are increasingly identified earlier in the disease course.

In our study, when we compared patients from the pre-COVID-19 (*n* = 47) and post-COVID-19 (*n* = 53) periods, no significant difference was observed in the etiological distribution. Although some studies in the literature have suggested that COVID-19 may lead to increased autoimmunity, inflammatory conditions and even immune-mediated hepatitis in child population, through mechanisms such as autoantibody formation, cytokine release, alterations in lymphocyte subsets, our findings did not reveal any change in etiological patterns supporting this association [41–43].

Given the inherent limitations of single-center, retrospective study, future multicenter collaborations are warranted to more accurately capture epidemiological trends and enhance the generalizability of findings in patients with FUO.

## Conclusion

Our findings illustrate that the etiological profile of FUO is shifting, with inflammatory diseases now surpassing infections in tertiary care settings. PET-CT can facilitate early identification of high-risk patients, particularly those with malignancies. Atypical presentations, such as thyroiditis or relapsing fever, require careful evaluation, and clinical features like arthralgia and anemia remain valuable diagnostic clues. Given its high diagnostic yield and impact on clinical decision-making, PET-CT should be considered early in the diagnostic algorithm, especially in cases with suspected malignancy or infection.

Subgroup-specific considerations—such as the absence of autoimmune diseases in HIV-associated FUO and the predominance of drug-induced pneumonitis in immunosuppressed patients—highlight the need for tailored diagnostic strategies. Also relapsing FUO remains a diagnostic challenge and warrants further investigation in future prospective studies.

Despite technological advances, invasive procedures such as biopsy continue to play a central role in unresolved cases. In regions where TB remains endemic, clinicians should maintain a high index of suspicion for extrapulmonary TB, particularly when initial workup is inconclusive.

## Data Availability

The datasets generated and/or analyzed during the current study are not publicly available due to patient confidentiality and institutional data protection policies.

## Abbreviations

AIDS: Acquired Immunodeficiency Syndrome
ALP: Alkaline Phosphatase
ALT: Alanine Aminotransferase
AST: Aspartate Aminotransferase
AUC: Area Under the Curve
CMV: Cytomegalovirus
CRP: C-reactive Protein
CVD: Collagen Vascular Disease
ESR: Erythrocyte Sedimentation Rate
FUO: Fever of Unknown Origin
GGT: Gamma-glutamyl Transferase
HIV: Human Immunodeficiency Virus
IRIS: Immune Reconstitution Inflammatory Syndrome
MAC: Mycobacterium avium Complex
PCR: Polymerase Chain Reaction
PET-CT: Positron Emission Tomography–Computed Tomography
ROC: Receiver Operating Characteristic
TB: Tuberculosis
USG: Ultrasonography
VDRL: Venereal Disease Research Laboratory
